# The Potential Role of Salivary NT-proBNP in Heart Failure

**DOI:** 10.3390/life13091818

**Published:** 2023-08-28

**Authors:** Aidonis Rammos, Aris Bechlioulis, Petros Kalogeras, Chris J. Watson, Pietro Salvo, Tommaso Lomonaco, Olga Kardakari, Evanthia E. Tripoliti, Yorgos Goletsis, Dimitris I. Fotiadis, Christos S. Katsouras, Lampros K. Michalis, Katerina K. Naka

**Affiliations:** 1 2nd Department of Cardiology, Faculty of Medicine, School of Health Sciences, University of Ioannina & University Hospital of Ioannina, 45110 Ioannina, Greecepkalog90@yahoo.com (P.K.); o.kardakari@uoi.gr (O.K.);; 2 Wellcome-Wolfson Institute for Experimental Medicine, Queen’s University Belfast, Belfast BT9 7BL, UK; chris.watson@qub.ac.uk; 3 UCD Conway Institute, School of Medicine, University College Dublin, 4 Dublin, Ireland; 4 Institute of Clinical Physiology, Italian National Research Council, Via G. Moruzzi 1, 56124 Pisa, Italy; 5 Department of Chemistry and Industrial Chemistry, University of Pisa, 56124 Pisa, Italy; tommaso.lomonaco@unipi.it; 6 Department of Biomedical Research, Institute of Molecular Biology and Biotechnology, FORTH, 45110 Ioannina, Greeceyorgos.gol@gmail.com (Y.G.); dimitris.fotiadis30@gmail.com (D.I.F.); 7 Department of Economics, University of Ioannina, 45110 Ioannina, Greece; 8 Unit of Medical Technology and Intelligent Information Systems, University of Ioannina, 45110 Ioannina, Greece

**Keywords:** acute heart failure, chronic heart failure, heart failure diagnosis, saliva NT-proBNP, salivary biomarkers

## Abstract

Background: Serum natriuretic peptides (NPs) have an established role in heart failure (HF) diagnosis. Saliva NT-proBNP that may be easily acquired has been studied little. Methods: Ninety-nine subjects were enrolled; thirty-six obese or hypertensive with dyspnoea but no echocardiographic HF findings or raised NPs served as controls, thirteen chronic HF (CHF) patients and fifty patients with acute decompensated HF (ADHF) requiring hospital admission. Electrocardiogram, echocardiogram, 6 min walking distance (6MWD), blood and saliva samples, were acquired in all participants. Results: Serum NT-proBNP ranged from 60–9000 pg/mL and saliva NT-proBNP from 0.64–93.32 pg/mL. Serum NT-proBNP was significantly higher in ADHF compared to CHF (*p* = 0.007) and in CHF compared to controls (*p* < 0.05). There was no significant difference in saliva values between ADHF and CHF, or between CHF and controls. Saliva and serum levels were positively associated only in ADHF patients (R = 0.352, *p* = 0.012). Serum NT-proBNP was positively associated with NYHA class (R = 0.506, *p* < 0.001) and inversely with 6MWD (R = −0.401, *p* = 0.004) in ADHF. Saliva NT-proBNP only correlated with age in ADHF patients. Conclusions: In the current study, saliva NT-proBNP correlated with serum values in ADHF patients, but could not discriminate between HF and other causes of dyspnoea. Further research is needed to explore the value of saliva NT-proBNP.

## 1. Introduction

Heart failure (HF) is a clinical syndrome characterized by symptoms, such as dyspnoea, orthopnoea, paroxysmal nocturnal dyspnoea, impaired exercise tolerance, and signs of pulmonary and/or systemic venous congestion, that are associated with structural and/or functional cardiac abnormalities that result in a reduced cardiac output and/or elevated intracardiac pressures at rest or during stress [1,2]. The etiology of HF is variable with coronary artery disease, hypertension, arrhythmias, valvular and myocardial disease, toxic damage from recreational substance use and chemotherapy, infiltrative diseases, and genetic abnormalities being the commonest causes [1].

According to the latest guidelines by the European Society of Cardiology (ESC) on HF, serum natriuretic peptides (NPs), BNP and/or NT-proBNP, are the only biomarkers with an established role in HF diagnosis. Diagnosis of HF is based on elevated levels of NPs (BNP > 35 pg/mL, NT-proBNP > 125 pg/mL), while in cases these are unavailable or if HF is strongly suspected, an echocardiogram should be performed [1]. However, in order to measure NPs, venous blood sampling is needed, a procedure that requires clinical skills and may be difficult to perform in an acute event and in some clinical settings.

Saliva, a body fluid containing several proteins that might be used as potential biomarkers, has the advantage of being easily collected in a non-invasive way, while it can be applied in various point-of-care (PoC) devices [3,4], as a tool to improve patient care by providing a rapid and actionable result near the patient [5]. Saliva has been mainly used in the detection of oral cancer and oral inflammation [6,7], while biomarkers associated with coronary artery disease such as CRP, CK-MB, sCD40 ligand, hs-cTnT, cTnI [8,9,10,11,12,13] and HF such as galectin-3 (Gal-3), cortisol, TNF, interleukins 6 and 10, CRP, BNP and NT-proBNP, have also been detected in saliva [14,15,16,17,18,19,20]. Despite the extensive literature on serum NPs, only three studies have measured salivary NPs [15,21,22]. Whether these correlate to serum NP levels or may be used in HF diagnosis and management has been little studied. 

In the current study, salivary and serum NT-proBNP levels were prospectively measured in patients with stable chronic HF (CHF), acute decompensated HF (ADHF) admitted to hospital, and subjects with hypertension or obesity who presented with symptoms like dyspnoea but were not diagnosed with HF. We aimed to compare the biomarker’s saliva and serum values between groups, and to study their correlation as well as their potential association with clinical risk factors (i.e., chronic kidney disease, obesity, etc.), markers of functional capacity, and echocardiographic indices of intra-cardiac pressures. 

## 2. Materials and Methods

The study was conducted according to the guidelines of the Declaration of Helsinki, and approved by the Institutional Review Board of the University Hospital of Ioannina. Informed consent was obtained from all subjects who participated in the study.

### 2.1. Study Participants

The study included 99 participants who were enrolled from November 2020 until June 2021. Thirty-six subjects with symptoms suggestive of HF but no evidence of ventricular dysfunction on echocardiography and/or raised serum NPs, were enrolled from the General Cardiology out-patient clinic of the Ioannina University Hospital and served as controls. Their main medical condition was obesity or hypertension. Obesity was defined as body mass index (BMI, calculated as weight in kilograms divided by height in meters squared) greater than 30 kg/m^2^. Hypertension was defined as a consistent increase in systolic and or diastolic blood pressure (SBP ≥ 140 mmHg ± DBP ≥ 90 mmHg) or >1 month use of antihypertensive medications. Thirteen patients, with previously known stable CHF with reduced, mildly reduced, or preserved left ventricular ejection fraction (LVEF) of any underlying etiology but with no decompensation in the last 6 months [1], were enrolled from our HF out-patient clinic of the Ioannina University Hospital. Finally, 50 consecutive patients who were admitted in the Second Cardiology Department of the Ioannina University Hospital with a diagnosis of ADHF (newly diagnosed or known HF) were enrolled. 

Exclusion criteria were: patients currently positive to SARS-CoV-2 or recently recovered from COVID-19, aged less than 18 years of age, unable or unwilling to give informed consent, cognitive, mental or psychiatric disorders, alcohol or drug abuse, pregnant or breast-feeding female patients, patients on vegan and/or vegetarian diets, patients with severe co-morbidities such as severe chronic obstructive pulmonary disease (e.g., patients on oxygen therapy or nebulizers at home), uncontrolled dysthyroidism, decompensated diabetes mellitus, chronic inflammatory intestinal diseases, severe or active rheumatological disease, active oncological disease on current or previous therapy of less than 1 year, liver failure and severe kidney disease [estimated glomerular filtration rate (eGFR) less than 30 mL/min/1.73 m^2^]. 

### 2.2. Study Procedures

All enrolled subjects were subjected to a nasopharyngeal or pharyngeal test for SARS-CoV-2 with all the appropriate precautions. A detailed medical history was obtained including all medications received, family history, patients’ habits and lifestyle. Afterwards, a thorough physical examination was performed. Blood samples were collected with standard venipuncture procedures for routine laboratory investigations. NT-proBNP was measured using a POC device (Cobas h 232, Roche Diagnostics, GmbH, Mannheim, Germany). After blood sampling, saliva sample collection followed as described in detail below. Subsequently, a 12-lead electrocardiogram and a transthoracic echocardiogram were recorded. A 6 min walk test was performed for all patients who were able to walk in the corridor. All patients were studied in the morning after an overnight fasting. ADHF patients were studied as early as their medical condition allowed within the first 24 h of their admission.

### 2.3. Saliva Sample Collection

Stimulated morning saliva samples were collected with a commercially available kit (SalivaBio Oral Swab Device, Salimetrics LLC, Carlsbad, CA, USA) between 8 a.m. and 10 a.m. All enrolled subjects had no clinical signs of oral inflammation, no medical history of oral cancer or inflammation, and they did not have any dental work or treatment during the study period. They were also asked to refrain from eating, drinking, smoking, chewing gum, and oral hygiene practices for at least 2 h prior to saliva collection. The samples were then centrifuged at 5000× *g* rotations per minute (rpm) for 6 min, at 4 °C. In severely decompensated HF patients, the procedure might be repeated two or three times until an adequate quantity (1 mL) of saliva was collected due to reduced saliva production. Samples were stored at −20 °C until their analysis with ELISA kits (NT-proBNP ELISA SK-1204, Biomedica Medizinprodukte GmbH, Vienna, Austria) as reported elsewhere [4].

### 2.4. Statistical Analysis

Normal distribution of values was evaluated for all variables using the Kolmogorov—Smirnoff test. Continuous data are presented as mean ± standard deviation or median values (interquartile range), while dichotomous data are presented as number (percentage). Comparisons among the three groups of chronic patients (obese vs. hypertensive vs. CHF) were made using the Fischer x^2^ test for dichotomous variables and one-way ANOVA test (with a Bonferroni correction for post-hoc comparisons). Comparisons between the two groups of HF patients (CHF vs. ADHF) were made using the Fischer x^2^ test for dichotomous variables and the student’s *t*-test or Mann—Whitney U test for continuous variables. Spearman and Pearson’s correlation coefficients were assessed to detect potential associations of serum and saliva NT-proBNP levels with other parameters. A two-tailed *p*-value < 0.05 was used to determine significant associations. All analyses were performed with the software IBM SPSS Statistics version 21 (IBM, Armonk, NY, USA).

## 3. Results

### 3.1. Comparison of Obese vs. Hypertensive vs. Chronic HF Patients

The descriptive data of patients in the three groups are shown in Table 1. 

**Figure 1:**
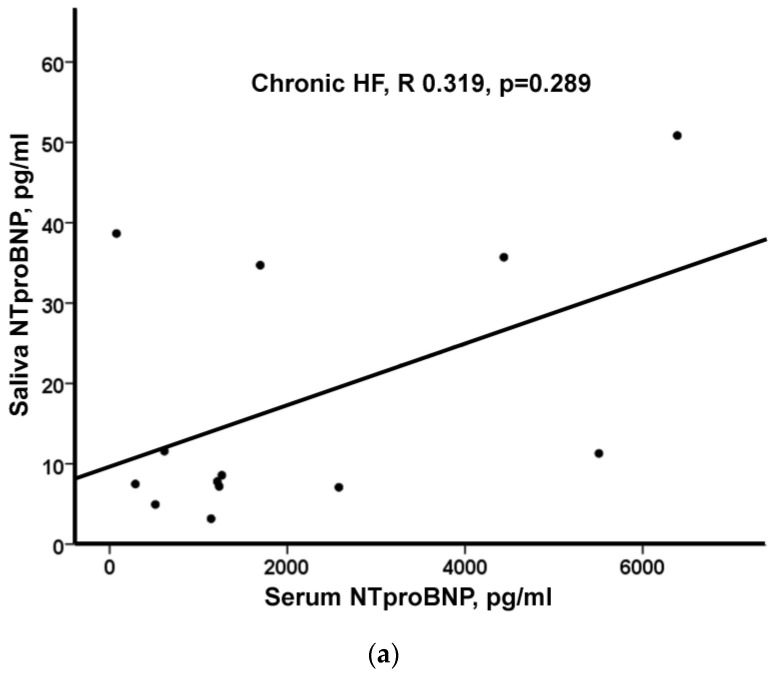

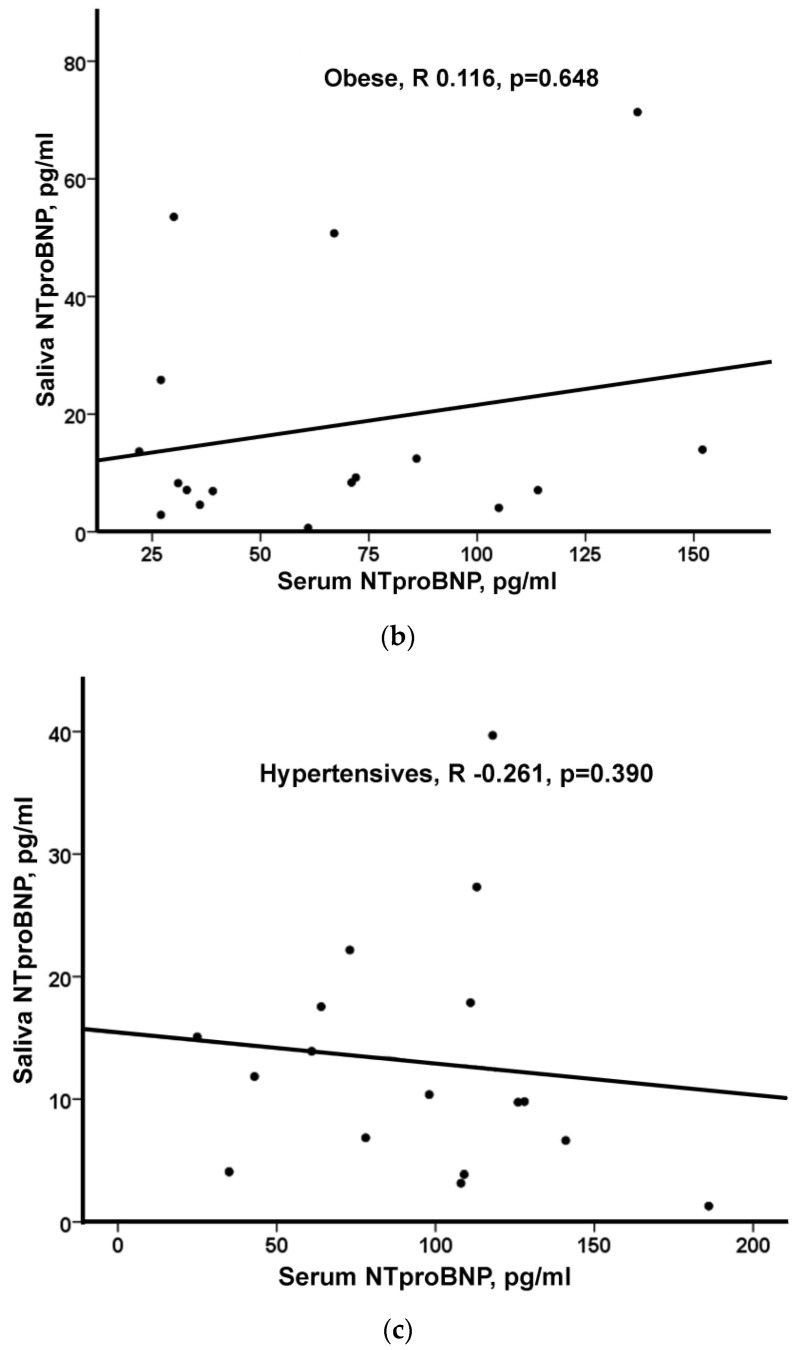
(**a**) Correlation between serum and saliva levels of NT-proBNP in chronic HF (CHF) patients (n = 13). (**b**) Correlation between serum and saliva levels of NT-proBNP in obese patients (n = 18). (**c**) Correlation between serum and saliva levels of NT-proBNP in hypertensive patients (n = 18).

Hypertensive and CHF patients were older compared to obese patients (*p* < 0.05 for both). There were various differences in the prevalence of cardiovascular risk factors and cardiovascular disease, as well as the use of medications among the three groups. However, it should be noted that many patients with hypertension received medications prescribed for HF [e.g., angiotensin receptor blockers, beta blockers, mineralocorticoid receptor antagonists, and thiazide diuretics (67%, 39%, 6%, and 33%, respectively)] (Table 1). Hypertensive and obese patients walked a greater 6 min walk distance (6MWD) compared to CHF patients (*p* < 0.05 for both) and had a higher value of LVEF (*p* < 0.05 for both). Serum NT-proBNP levels were higher in CHF compared to both obese and hypertensive patients [1232 pg/mL (566, 3509) vs. 64 pg/mL (<60, 93) vs. 106 pg/mL (63, 120), respectively (*p* < 0.05)] but there was no significant difference in saliva NT-proBNP levels among the three groups of patients [8.6 pg/mL (7.1, 35.2) vs. 8.8 pg/mL (6.3, 22.7) vs. 11.1 pg/mL (6.0, 17.6), respectively (*p* = 0.969)]. No significant correlation between serum and saliva levels of NT-proBNP was observed in any of the three groups (Figure 1).

### 3.2. Comparison of Chronic HF vs. Acute Decompensated HF Patients

No significant differences were found in age, gender, risk factors, and cardiovascular history between the two groups (*p* = NS for all, Table 2). 

**Figure 2:**
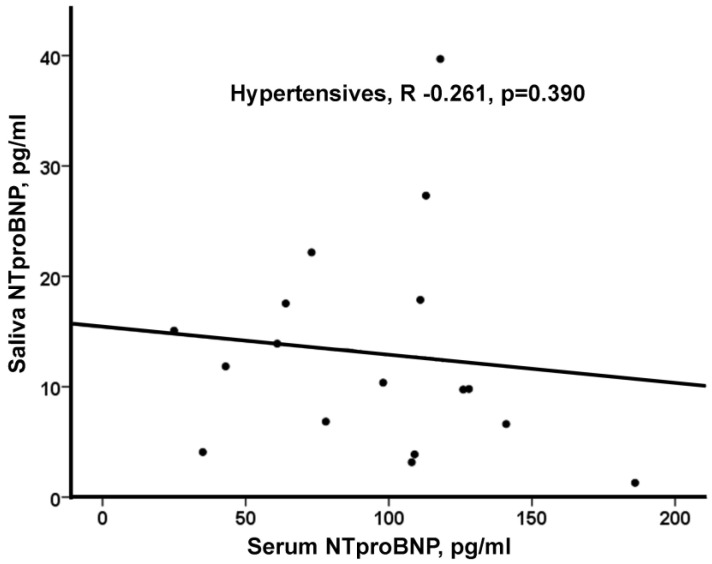
Correlation between serum and saliva levels of NT-proBNP for acute decompensated HF (ADHF) patients (n = 50).

Higher NYHA class and lower 6MWD values were observed in ADHF compared to CHF patients (*p* < 0.05 for both, Table 2). Increased E/E’ and inferior vena cava diameter were found in ADHF vs. CHF patients (*p* < 0.05 for both, Table 2). Serum NT-proBNP was found to be significantly higher in ADHF compared to CHF patients [4706 pg/mL (1237, 8438) vs. 1232 pg/mL (566, 3509), respectively (*p* = 0.007)] while there was no significant difference in saliva NT-proBNP levels between the two groups, [15.1 pg/mL (10.4, 30.6) vs. 8.6 pg/mL (7.1, 35.2), respectively (*p* = 0.122)] (Table 2). Saliva and serum levels of NT-proBNP were positively associated only in ADHF patients (R = 0.352, *p* = 0.012, Figure 2). 

Significant bivariate associations of saliva and serum NT-proBNP levels with various studied parameters are shown in Table 3. Saliva NT-proBNP was not associated with either NYHA class or 6MWD in any of the two groups, while serum NT-proBNP levels were positively associated with NYHA class (R = 0.506, *p* < 0.001) and inversely with 6MWD (R = −0.401, *p* = 0.004) only in ADHF patients (Table 3).

## 4. Discussion

In the current study, salivary NT-proBNP levels were found to be higher in ADHF patients compared to CHF patients as well as in CHF patients compared to obese and hypertensive patients, although these differences in saliva NT-proBNP levels among the groups of studied patients did not prove to be statistically significant. Moreover, saliva and serum NT-proBNP levels were significantly, albeit weakly, associated only in patients with ADHF. 

To our knowledge, this was the first clinical study that compared saliva and serum NT-proBNP in these three groups of patients (non-HF controls, CHF, and ADHF). A previous study in hospitalized HF patients has shown no association between serum and saliva BNP levels [15]. In that study, patients had a functional capacity of NYHA stages I-III, with 50% of them classified as NYHA I-II [15]. An important difference that needs to be noted between the two studies was that our study included patients with worse NYHA classes [i.e., 61% of CHF patients had a functional class of NYHA III and 98% of patients with ADHF had a functional class of III-IV, with 38% of them classified as NYHA IV]. Another study measured salivary BNP and serum NT-proBNP in controls, CHF, and ADHF patients [21]. In that study, saliva NP levels were shown to be higher in HF populations (chronic and acute) compared with non-HF patients, while a significant correlation between salivary BNP and plasma NT-proBNP concentrations (Pearson correlation, *p* < 0.001, r = 0.459) was also reported [21]. The third study that measured salivary NPs in CHF patients (NYHA III, reduced LVEF less than 40%) reported significantly higher saliva NT-proBNP levels in CHF patients compared to healthy controls, but failed to show an association between saliva and serum NT-proBNP levels in both groups [22]. The discrepancy in results between our study and previously published ones, may be attributed to differences in the populations studied, the type of NP measured (BNP vs. NT-proBNP), the method of NPs measurement in blood but mainly in saliva, as well as the sample storage conditions. Of note, a common finding in all studies concerning salivary NPs (including the current study) was the very low concentration of NPs in saliva [15,21,22].

Interestingly, we found a significant but weak positive association of serum and saliva NT-proBNP only in the ADHF patients (R = 0.352, *p* = 0.012), a finding that deserves further evaluation and validation as saliva may be a potential easily acquired biological fluid that can be used for ADHF diagnosis. The only study reporting a relationship of the biomarker values in the two biological fluids (saliva vs. serum) found a ratio of approximately 1:200 in ADHF patients, indicating that saliva may not be appropriate for NP detection [22]. In addition, there is no established pathophysiological background for the excretion of NPs in saliva in HF patients. The poor correlation between biomarker levels in saliva and serum may suggest an impaired transportation of NT-proBNP from the blood circulation into the saliva in HF patients, with a less efficient mechanism of converting proBNP (precursor molecule) by furin convertase into NT-proBNP, since its enzymatic activity in saliva is inhibited by histatins, preventing in situ generation of salivary NT-proBNP [23,24].

Our study investigated extensively potential associations of saliva NT-proBNP with echocardiographic indices of HF and other important clinical parameters, such as age, renal function, and 6MWD. Patients were symptom-matched rather than age-matched, which could explain some differences in saliva values or clinical parameters (such as the 6MWD). However, we preferred our populations to be symptom-matched, as it is difficult to find sexagenarians and septuagenarians without other health problems, and mark any differentiations among the age groups. 

Serum NT-proBNP values have been described as a weak discriminator of NYHA classification, since an overlap has been reported especially between NYHA I and II classes [25]. The serum biomarker has also been inversely correlated with 6MWD in HF patients [26]. In our study, we found that serum NT-proBNP was associated with functional capacity (NYHA class and 6MWD) especially in ADHF patients, as well as with other clinical variables in HF patients. However, none of these associations were observed with the saliva biomarker levels. Similar to our results, another study reported no significant association of saliva NT-proBNP with NYHA class [9]. The very low concentration of NPs in saliva probably does not allow for associations with other clinical variables to be demonstrated. 

The potentially valuable role of saliva biomarkers (such as NT-proBNP) in HF diagnosis and management, instead of using the serum/plasma biomarkers, is an interesting concept that has been introduced in various recent studies [3]. HF patients need frequent blood sampling either for diagnostic or treatment monitoring purposes. Thus, measuring salivary biomarkers, especially using a POC device, could prove to be extremely useful in clinical decision making, in out-of-hospital healthcare settings, primary health care, or in remote locations [27,28]. Although the current study did not demonstrate a diagnostic role of saliva NT-proBNP, the association between saliva and serum levels of NT-proBNP in ADHF patients may suggest that saliva NT-proBNP could potentially be used for monitoring of treatment effects in patients with acute HF (in-hospital or early post-discharge), using serial measurements. 

It should be stated that different methods were used for the determination of NT-proBNP in serum and saliva, as there is no standardized method for saliva NT-proBNP measurement. The kit used for serum measurements uses biotinylated polyclonal anti-NT-proBNP antibodies and gold-labeled monoclonal anti-NT-proBNP antibodies, while for saliva the kit uses AA 32–57 (polyclonal sheep anti-human NT-proBNP) as capture antibody and AA 8–29 (HRP-labeled polyclonal sheep anti-human NT-proBNP) as detection antibody. This may have had an impact on correlation. As a matter of fact, from the studies measuring saliva NPs, only one used the same method for biomarker measurement [15,21,22]. 

In our study, we chose to measure NT-proBNP as this is a more stable molecule than BNP, with a longer half-life (60–120 min compared to approximately 18–20 min for BNP) [29]; the slower clearance of NT-proBNP from the blood may have possibly allowed for a transportation of the molecule into the saliva through various routes, but mainly via the gingival crevicular fluid [22,30]. Moreover, the collection procedures have an impact on the concentration of biomolecules in saliva, and stimulated samples have low content of proteins and peptides. The reason we decided to use stimulated saliva was to obtain the proper amount of sample that was required for the chemical analysis. It was noticed, during the sample collection, that the severely decompensated HF patients could not produce the required amount of saliva (1 mL) and the procedure for saliva collection might be repeated two or three times. Finally, saliva also contains other molecules, e.g., electrolytes (sodium, potassium, etc.) that are extremely important in the management of HF patients [31,32] and future studies are needed to evaluate their usability in a clinical setting. A potential combination of these biomarkers with NT-proBNP might be useful for monitoring HF therapy and management guidance.

## 5. Limitations

The number of subjects enrolled in this study was rather limited. Although this group of 99 patients compares favorably with previous studies using saliva NPs, larger studies are needed to draw definite conclusions. Unfortunately, the SARS-CoV-2 pandemic was unavoidably an important limiting factor in a study based on saliva samples. Another limiting factor is the fact that most patients with hypertension received medications prescribed for HF [e.g., angiotensin receptor blockers (67% of patients), beta blockers (39%), mineralocorticoid receptor antagonists (6%), and thiazide diuretics (33%)]; the use of these medications may have led to a reduction in their NP levels and misclassification of subjects. Saliva quality and quantity are affected by several other medical conditions and treatments, as well as the patient’s psychological state [33,34,35], while the use of different methods for NT-proBNP determination in serum and saliva may have an impact on correlation. Finally, in severely decompensated HF patients, the procedure for saliva collection might be repeated to acquire the proper amount of saliva.

## 6. Conclusions

The results of the current study show that, although saliva NT-proBNP levels correlated with serum values in patients with ADHF, they could not discriminate between HF and other causes of dyspnoea. Further research is needed to explore the value of saliva NT-proBNP, as an HF biomarker that can be acquired easily and non-invasively using saliva, especially to guide management in patients admitted with acute HF.

## Figures and Tables

**Table 1:** Demographic and clinical data of obese, hypertensive, and chronic HF (CHF) patients.

	CHF, n = 13	Obese, n = 18	Hypertensive, n = 18	*p*-Value
Age, years, median (IQR)	75 (64, 78) ^†^	48 (42, 58)	66 (57, 74) ^†^	<0.001
Male gender, n (%)	7 (54)	8 (44)	9 (50)	0.870
CV risk factors, n (%)				
Hypertension	10 (77)	5 (28)	18 (100)	<0.001
Dyslipidemia	11 (85)	7 (39)	15 (83)	0.005
Diabetes	4 (31)	0 (0)	3 (17)	0.051
Obesity	4 (31)	18 (100)	6 (33)	<0.001
Smoking	1 (8)	6 (33)	3 (17)	0.265
History, n (%)				
CAD	5 (39)	0 (0)	3 (17)	0.017
Stroke/TIA	1 (8)	0 (0)	1 (6)	0.522
PAD	1 (8)	0 (0)	0 (0)	0.243
CKD	3 (23)	1 (6)	1 (6)	0.012
AF	6 (46)	1 (6)	1 (6)	0.003
Medications, n (%)				
ACEi	3 (23)	0 (0)	1 (6)	0.06
ARBs	4 (31)	1 (6)	12 (67)	0.001
CCB	6 (46)	2 (11)	10 (56)	0.016
Statins	9 (69)	3 (17)	15 (83)	<0.001
B-blockers	8 (62)	4 (22)	7 (39)	0.086
MRA	5 (39)	1 (6)	1 (6)	0.015
Loop diuretics	8 (62)	1 (6)	0 (0)	<0.001
Thiazide diuretics	2 (15)	0 (0)	6 (33)	0.026
SBP, mmHg	133 ± 16	139 ± 14	149 ± 22 *	0.045
DBP, mmHg	67 ± 11	84 ± 11 *	87 ± 13 *	<0.001
BMI, kg/m^2^	28.6 ± 4.0 ^†^	35.1 ± 3.5	29.9 ± 4.4 ^†^	<0.001
6MWD, m	362 ± 165	531 ± 121 *	492 ± 103 *	0.002
Hb, g/dL	13.3 ± 2.3	14.3 ± 1.9	14.3 ± 1.2	0.220
Glucose, mg/dL	98 (90, 157)	94 (90, 106)	101 (94, 107)	0.421
eGFR, mL/min/1.73 m^2^	64.5 ± 17.4	82.2 ± 22.9 *	80.0 ± 13.5	0.026
TCHOL, mg/dL	184 ± 52	219 ± 31	180 ± 53 ^†^	0.032
HDL, mg/dL	48 ± 13	55 ± 10	55 ± 13	0.226
TRG, mg/dL	129 ± 63	113 ± 44	109 ± 42	0.505
LDL, mg/dL	111 ± 43	142 ± 33	104 ± 40 ^†^	0.032
Echocardiography				
LVEF, %	45 ± 15	60 ± 2 *	60 ± 3 *	<0.001
LVIDD, mm	52 ± 10	48 ± 5	43 ± 6 *	0.004
RWT	0.47 ± 0.13	0.42 ± 0.09	0.54 ± 0.12 ^†^	0.011
LVH, n (%)	6 (46)	4 (22)	7 (39)	0.345
E, cm/s	99 ± 27	76 ± 19 *	69 ± 14 *	0.001
E/E’	12.0 (8.8, 13.5)	7.5 (6.8, 9.0)	7.0 (6.9, 9.0)	<0.001
TRVmax, m/s	2.6 ± 0.7	2.1 ± 0.6	1.9 ± 0.5 *	0.009
Serum NT-proBNP, pg/mL, median (IQR)	1232 (566, 3509)	64 (<60, 93)	106 (63, 120)	<0.001
Saliva NT-proBNP, pg/mL median (IQR)	8.6 (7.1, 35.2)	8.8 (6.3, 22.7)	11.1 (6.0, 17.6)	0.969

* post-hoc analysis *p* < 0.05 vs. chronic HF; ^†^ post-hoc analysis *p* < 0.05 vs. obese. Abbreviations: 6MWD, six minute walk distance; ACEi, angiotensin converting enzyme inhibitors; AF, atrial fibrillation; ARBs, angiotensin II receptor blockers; ARNI, angiotensin receptor-neprilysin inhibitor; BMI, body mass index; CAD, coronary artery disease; CCB, calcium channel blockers; CKD, chronic kidney disease; CV, cardiovascular; DBP, diastolic blood pressure; eGFR, estimated glomerular filtration rate; LAVI, left atrial volume index; LVEF, left ventricle ejection fraction; LVH, left ventricular hypertrophy; LVIDD, left ventricular internal end diastolic diameter; MRA, mineralocorticoid receptor antagonist; NYHA, New York Heart Association; PAD, peripheral artery disease; RWT, regional wall thickness; SBP, systolic blood pressure; TIA, transient ischemic attack.

**Table 2:** Demographic and clinical data of chronic HF (CHF) and acute decompensated HF (ADHF) patients.

	Chronic HF, n = 13	ADHF, n = 50	*p*-Value
Age, years, median (IQR)	75 (64, 78)	76 (63, 83)	0.981
Male gender, n (%)	7 (54)	35 (70)	0.329
CV risk factors, n (%)			
Hypertension	10 (77)	43 (86)	0.417
Dyslipidemia	11 (85)	38 (76)	0.714
Diabetes	4 (31)	25 (50)	0.349
Obesity	4 (31)	11 (22)	0.489
Smoking	1 (8)	6 (12)	0.882
History, n (%)			
CAD	5 (39)	22 (44)	0.764
Stroke/TIA	1 (8)	6 (12)	0.660
PAD	1 (8)	4 (8)	0.971
CKD	3 (23)	23 (46)	0.207
AF	6 (46)	29 (58)	0.537
Medications, n (%)			
ACEi	3 (23)	8 (16)	0.549
ARBs	4 (31)	11 (22)	0.508
ARNI	1 (8)	5 (10)	0.801
CCB	6 (46)	8 (16)	0.020
Statins	9 (69)	29 (58)	0.538
B-blockers	8 (62)	34 (68)	0.660
MRA	5 (39)	26 (52)	0.536
Loop diuretics	8 (62)	37 (74)	0.376
Anticoagulation	5 (39)	31 (62)	0.208
Antiplatelets	5 (39)	15 (30)	0.559
SBP, mmHg	133 ± 16	130 ± 25	0.628
DBP, mmHg	67 ± 11	70 ± 14	0.446
HR, bpm	73 ± 20	79 ± 16	0.265
BMI, kg/m^2^	28.6 ± 4.0	27.2 ± 4.8	0.331
6MWD, m	362 ± 165	263 ± 151	0.045
NYHA class			
I	1 (8)	0 (0)	
II	3 (23)	1 (2)	
III	8 (61)	30 (60)	
IV	1 (8)	19 (38)	0.003
QRS duration, msec	118 (90, 159)	140 (99, 158)	0.535
WBC	7238 ± 2059	8499 ± 2832	0.139
Hb, g/dL	13.3 ± 2.3	12.5 ± 2.2	0.261
Glucose, mg/dL	98 (90, 157)	138 (106, 168)	0.036
Urea, mg/dL	51 (39, 80)	73 (54, 101)	0.045
eGFR, mL/min/1.73 m^2^	64.5 ± 17.4	55.1 ± 18.5	0.107
K+	4.05 ± 0.41	4.24 ± 0.52	0.216
Na+	139 (138, 141)	138 (135, 140)	0.189
TCHOL, mg/dL	184 ± 52	154 ± 36	0.016
HDL, mg/dL	48 ± 13	43 ± 13	0.281
TRG, mg/dL	129 ± 63	125 ± 65	0.844
LDL, mg/dL	111 ± 43	85 ± 29	0.013
Echocardiography			
LVEF, %	45 ± 15	38 ± 19	0.206
LVIDD, mm	52 ± 10	57 ± 12	0.176
RWT	0.47 ± 0.13	0.42 ± 0.16	0.366
LVH, n (%)	6 (46)	31 (62)	0.353
E, cm/s	99 ± 27	102 ± 42	0.775
E/E’	12.0 (8.8, 13.5)	15.0 (12.8, 16.0)	0.018
TRVmax, m/s	2.6 ± 0.7	3.0 ± 0.8	0.131
IVC diameter, mm	13 (10, 19)	23 (20, 25)	0.001
Serum NT-proBNP, pg/mL, median (IQR)	1232 (566, 3509)	4706 (1237, 8438)	0.007
Saliva NT-proBNP, pg/mL, median (IQR)	8.6 (7.1, 35.2)	15.1 (10.4, 30.6)	0.122

Abbreviations: 6MWD, six minute walk distance; ACEi, angiotensin converting enzyme inhibitors; AF, atrial fibrillation; ARBs, angiotensin II receptor blockers; ARNI, angiotensin receptor-neprilysin inhibitor; BMI, body mass index; CAD, coronary artery disease; CCB, calcium channel blockers; CKD, chronic kidney disease; CV, cardiovascular; DBP, diastolic blood pressure; eGFR, estimated glomerular filtration rate; IVC, inferior vena cava; LAVI, left atrial volume index; LVEF, left ventricle ejection fraction; LVH, left ventricular hypertrophy; LVIDD, left ventricular internal end diastolic diameter; MRA, mineralocorticoid receptor antagonist; NYHA, New York Heart Association; PAD, peripheral artery disease; RWT, regional wall thickness; SBP, systolic blood pressure; TIA, transient ischemic attack.

**Table 3:** Serum and saliva NT-proBNP (pg/mL) association with other characteristics in chronic HF patients and acute decompensated HF patients.

Chronic HF Patients, n = 13			
Serum NT-proBNP (pg/mL)		Saliva NT-proBNP (pg/mL)	
CAD	R = 0.676, *p* = 0.011	NYHA class	R = 0.054, *p* = 0.862
Loop diuretics	R = 0.634, *p* = 0.020	6MWD, m	R = −0.429, *p* = 0.143
eGFR, mL/min/1.73 m^2^	R = −0.731, *p* = 0.005		
NYHA class	R = 0.533, *p* = 0.061		
6MWD, m	R = −0.250, *p* = 0.409		
**ADHF patients, n = 50**			
**Serum NT-proBNP (pg/mL)**		**Saliva NT-proBNP (pg/mL)**	
SBP, mmHg	R = −0.286, *p* = 0.044	Age, years	R = −0.317, *p* = 0.025
eGFR, mL/min/1.73 m^2^	R = −0.347, *p* = 0.014	NYHA class	R = 0.224, *p* = 0.119
RWT	R = −0.302, *p* = 0.033	6MWD, m	R = −0.222, *p* = 0.125
E/E’	R = 0.404, *p* = 0.005		
IVC diameter, mm	R = 0.401, *p* = 0.004		
NYHA class	R = 0.506, *p* < 0.001		
6MWD, m	R = −0.401, *p* = 0.004		

Abbreviations: 6MWD, six minute walk distance; CAD, coronary artery disease; eGFR, estimated glomerular filtration rate; IVC, inferior vena cava; NYHA, New York Heart Association; RWT, regional wall thickness; SBP, systolic blood pressure.

## Data Availability

All data supporting reported results can be found on PubMed. The details are provided in References.
